# How important is importance for prospective memory? A review

**DOI:** 10.3389/fpsyg.2014.00657

**Published:** 2014-06-26

**Authors:** Stefan Walter, Beat Meier

**Affiliations:** ^1^Institute of Psychology, University of BernBern, Switzerland; ^2^Center for Cognition, Learning, and Memory, University of BernBern, Switzerland

**Keywords:** intention, importance manipulation, strategic monitoring, automatic retrieval, motivation

## Abstract

Forgetting to carry out an intention as planned can have serious consequences in everyday life. People sometimes even forget intentions that they consider as very important. Here, we review the literature on the impact of importance on prospective memory performance. We highlight different methods used to manipulate the importance of a prospective memory task such as providing rewards, importance relative to other ongoing activities, absolute importance, and providing social motives. Moreover, we address the relationship between importance and other factors known to affect prospective memory and ongoing task performance such as type of prospective memory task (time-, event-, or activity-based), cognitive loads, and processing overlaps. Finally, we provide a connection to motivation, we summarize the effects of task importance and we identify important venues for future research.

## Introduction

Prospective memory refers to the ability to plan, retain and retrieve an intention as planned. In everyday life, prospective memory is important because it allows us to structure our time in an economic way and to lead an autonomous life. It can also affect our reputation and self-esteem, for example, one may be perceived as conscientious and well organized or as unreliable and unstructured. Typically, we have more than one active intention and often, one intention is more important than another one. For example, attending your own wedding will certainly be considered more important than—let us say—to pay a bill in time. Accordingly, the more important intention is more likely to be remembered. Nevertheless, it happens that somebody forgets an intention although it is really important. For example, recently the story of a man was in the news who was jailed because he staged a bomb hoax in a church after he realized that he had forgotten to book the church for his own wedding, a failure of prospective memory. This example illustrates that even a very important intention, that is, remember to book the church for the own wedding can fail. The purpose of this article is to review the literature on the impact of importance in prospective memory research.

## Importance of intentions

The importance of an intention is based on values, desires, goals, and their predicted consequences (cf. Baars and Mattson, 1981; Kvavilashvili and Ellis, 1996). Thus, whether an intention is important or not is based on subjective valuing. However, in experimental research, importance is typically induced by the experimenter. In prospective memory research, common manipulation-methods include *providing a reward* (or manipulating task-attractiveness), *relative importance instructions* (i.e., emphasizing the prospective memory task relative to other ongoing activities), *absolute importance instructions* (i.e., emphasizing the prospective memory task *per se*) or *providing social motives* to perform the prospective memory task (cf. Meacham and Singer, 1977; Kliegel et al., 2004; Einstein et al., 2005; Brandimonte et al., 2010). In the first part of this article, we will review the relevant studies investigating the effect of importance on prospective memory and classify them by these different methods to manipulate importance. We also take into account the role of the specific kind of prospective memory task, effects of cognitive loads and processing overlaps that may interact with task importance. The kind of prospective memory task is determined by the definition of the specific cue that triggers the retrieval of an intention. In a time-based prospective memory task the intention must be remembered at a particular time (e.g., attend a meeting tomorrow at 2 p.m.), in an event-based task it must be retrieved when a particular event happens (e.g., remember to buy bread when passing the grocery store), and in an activity-based task it must be remembered after completing a particular activity (e.g., attach a file after having written an email message). Cognitive loads refer to the available processing resources when an intention must be remembered. Processing overlaps refer to the relationship between the processing operations required for processing the ongoing task and for remembering the intention, that is, focal tasks represent high processing overlaps (McDaniel and Einstein, 2000; Meier and Graf, 2000; Einstein et al., 2005). In a second section, we will discuss the different results from the perspective of two theoretical frameworks of prospective memory research, *preparatory attentional and memory processes* theory (PAM; Smith, 2003) and the *multiprocess* model (McDaniel and Einstein, 2000), in order to clarify the underlying mechanisms of importance effects. Finally, we will discuss the connection of task importance to motivation, to provide an alternative perspective to the field.

### Reward and task-attractiveness

*Providing a reward* is a naturalistic way to induce importance and its effectiveness can be varied by the kind of reward, by the amount of reward or by manipulating the *attractiveness* (or *unattractiveness*) of the prospective memory task (i.e., the reward is the benefit of remembering the intention). For example, in an early study, Meacham and Singer (1977) used rewards as a method to increase importance. The prospective memory task was to send a letter to the researcher on 8 different days over a period of 8 weeks. In the experimental condition, participants were told that they would receive money for each letter they returned on time and that they had the additional chance to win in a lottery if they would send back the letter. In the control condition no such promise was made. The results showed that the former participants were more likely to return the letters on time. Moreover, more of them reported having used external reminders in order to remember the prospective memory task. Thus, promising a reward increased performance, changed the perceived importance and also the strategy to be successful in the prospective memory task (see Jeong and Cranney, 2009, for recent replications).

Sommerville et al. (1983) manipulated importance by varying the attractiveness of the to-be-remembered intention. They instructed children, aged 2-, 3-, and 4-years old, either to remember to buy candies or to remember to clean the dishes. The results showed that children remembered the more attractive tasks more often than the less attractive tasks, suggesting that the attractiveness of a task increased the importance and, as a consequence, the likelihood to remember (see also Aberle et al., 2010; Kliegel et al., 2010; Ślusarczyk and Niedźwieńska, 2013, for similar replications).

Field studies such as those by Meacham and Singer (1977) and Sommerville et al. (1983) have the advantage of high ecological validity. However, in naturalistic studies the cognitive processes involved to perform the prospective memory task cannot be easily verified in detail. In order to gain control over these factors, laboratory studies are necessary. Therefore, a milestone in prospective memory research was the introduction of a computer-based laboratory paradigm by Einstein and McDaniel (1990)—although prospective memory research roots in the everyday memory movement (Neisser, 1974). The prospective memory task was kept as simple as possible and involved just pressing a key on the computer keyboard when a particular event occurred (e.g., the presentation of a specific word on the computer screen). Prospective memory performance was measured as the proportion of correct responses to prospective memory targets. Moreover, the prospective memory task was embedded in an *ongoing task* (a short-term memory task). The advantage of this method is that ongoing task performance can be assessed (i.e., by calculating ongoing task accuracy or reaction times). In subsequent studies, performance in the ongoing task was compared to a control condition with an additional prospective memory task and performance with vs. without having to keep a prospective memory task in mind was considered as *monitoring cost* (cf. Smith and Bayen, 2004). Furthermore, the opportunity to measure monitoring costs enabled them to investigate the resource demands of prospective memory, that is, whether successful retrieval needs *attention allocation* (i.e., enhancing monitoring costs; cf. Smith, 2003; Smith and Bayen, 2004) or whether it occurs *automatically* (i.e., without monitoring costs; cf. McDaniel and Einstein, 2000; Meier et al., 2006b).

In a laboratory setting, Krishnan and Shapiro (1999) investigated the influence of a monetary reward on prospective memory performance with a version of the shopping task as the prospective memory task and an association task as the ongoing task (cf. Einstein and McDaniel, 1990, 1996; for a similar task setting). For the shopping task, participants were told that they work for a shopping service that purchases products for five different customers. They were provided with a target word which described each customer and which was intended as prospective memory target. The prospective memory task was to write the word *customer* beside of a target word when it occurred during the association task. For the latter, participants were told to write down every product they associated with a certain brand. Importance was manipulated by informing half of the participants that they would receive one Dollar when they remembered the intention. The other half of participants were given the standard prospective memory instructions (i.e., the control group). Overall, the results showed higher prospective memory performance for the group that was promised a reward. Moreover, these participants tried harder to remember the prospective memory target than the control group as indicated by self-report. This was taken as an indicator of higher strategic monitoring.

In a similar vein, Guajardo and Best (2000) used a controlled computer-based prospective memory task to investigate the influence of a reward on prospective memory performance in preschoolers. The children were shown easy-to-name pictures on a computer screen and afterwards they had to recall as many pictures as possible. The prospective memory task was to press a key whenever a specific target picture appeared (either “duck” or “house”). Half of the children were allowed to select a reward (e.g., goldfish crackers, goldfish pretzels, pennies, or fruit chews) and they were told that they would get one each time they correctly performed the prospective memory task. The target picture appeared six times. Surprisingly, the results showed no beneficial effect of promising a reward. Thus, this study did not support the hypothesis that importance affects prospective memory performance, at least not in preschoolers (see Kliegel et al., 2003; for similar results assessed with a student sample).

In summary, three of the reviewed studies showed enhanced prospective memory performance in a *reward* condition compared to a standard prospective memory instruction condition (Meacham and Singer, 1977; Krishnan and Shapiro, 1999; Jeong and Cranney, 2009), two other studies, however, showed no such effect (Guajardo and Best, 2000; Kliegel et al., 2003). Furthermore, higher prospective memory task attractiveness also enhanced prospective memory performance (Sommerville et al., 1983; Kliegel et al., 2010; Ślusarczyk and Niedźwieńska, 2013).

### Relative importance manipulation

In most of the laboratory studies in which the impact of importance on prospective memory performance was investigated participants were either instructed to prioritize the prospective memory task or to prioritize the ongoing task, thus, varying the *relative importance* of a task (e.g., Kliegel et al., 2001, 2004; Smith and Bayen, 2004; Loft and Yeo, 2007; Loft et al., 2008). This kind of importance manipulation has been referred to as *dual-task-prioritizing* (cf. Burgess, 2000). Moreover, prospective memory performance is impaired in situations, in which working memory load is high (Marsh and Hicks, 1998). A straight-forward explanation is that insufficient cognitive resources are available for the prospective memory task. For example, driving home in rush hour traffic is more likely to cause us to forget to buy groceries along the way than driving home on an empty road. In the laboratory, cognitive loads can be manipulated in several different ways. First, the difficulty of the ongoing task can be varied (e.g., Marsh et al., 2002; McNerney and West, 2007; Khan et al., 2008; West et al., 2011). Second, an additional ongoing task can be added, thus requiring divided attention (e.g., Kliegel et al., 2001, 2004; Khan et al., 2008). Third, loads can be manipulated by adding and varying the difficulty of a third ongoing task (Wang et al., 2006). Fourth, the prospective memory task can be embedded in an ongoing task environment that requires task switching (e.g., Marsh et al., 2002; McNerney and West, 2007). The difficulty of the prospective memory task can also be varied, for example by using multiple prospective memory targets (cf. Einstein et al., 2005; Cohen et al., 2008). Consequently, in cognitive loaded situations strategic monitoring for prospective memory targets is impeded. Thus, successfully remembering of an important intention in such situations requires an intentional or explicit prioritizing of the prospective memory task.

Kliegel et al. (2001) manipulated relative importance and cognitive loads in two experiments in which the ongoing task was to rate words either on concreteness, familiarity, pleasantness, or seriousness in both experiments. In Experiment 1, the prospective memory task was *time-based*, that is, participants were asked to press a key every 2 min. They were allowed to monitor time by checking a clock that appeared on the screen whenever they pressed a different key. The results showed more accurate prospective memory responses when the prospective memory task was emphasized. This result suggests that the relative importance of an intention affects performance in time-based prospective memory tasks. In Experiment 2, participants were instructed for an *event-based* task. They had to press a special key whenever a specific word appeared during the word task. In addition, the load of the ongoing task was manipulated by asking the participants to perform an additional auditory digit detection task. The results showed that importance did not affect performance and neither did the manipulation of ongoing task load. Similarly, the importance manipulation did not generally affect attention allocation as measured as number of missed ongoing task trials (see also Harrison and Einstein, 2010).

In a follow-up study, Kliegel et al. (2004) replicated the latter result with a very similar set-up. Particularly, in their second experiment, the prospective memory targets were defined as words that contained either the letter *g* or *q* (i.e., a *non-focal* target) rather than as a specific word (i.e., a focal target, cf. Einstein and McDaniel, 2005). In this experiment, the importance manipulation enhanced prospective memory performance. Moreover, the impact of the importance manipulation was stronger when ongoing task load was increased by an additional auditory digit detection task. Strategic monitoring was enhanced by importance as more ongoing task omissions occurred in both experiments when the prospective memory task was emphasized.

Smith and Bayen (2004) specifically focused on monitoring costs triggered by emphasizing either the relative importance of the prospective memory or the ongoing task. The ongoing task was a color-matching task, and the prospective memory task was to press a particular key whenever one of six specific target words occurred (i.e., a cognitive “loaded” prospective memory task). The results showed higher prospective memory performance and more monitoring costs when the instructions emphasized the prospective memory task.

Loft and Yeo (2007, Experiment 2) investigated relative importance and the relation between prospective memory targets and responses (i.e., a cue-content overlap, cf. Meier et al., 2006a). The latter was manipulated by presenting word pairs, which were either strongly or weakly associated (i.e., high or low associations). The first word was used as the prospective memory target and the prospective memory task was to press a specific key and to type the second word of the word-pair whenever a prospective memory target word occurred. The prospective memory task was embedded in a lexical decision task. The results showed higher prospective memory performance when importance was emphasized and no influence of target-response association (i.e., cue-content overlap). Monitoring costs were higher in the condition that emphasized the prospective memory task and in the condition with weak target-response association (i.e., low cue-content overlap).

Loft et al. (2008, Experiment 2) also investigated whether the effect of relative importance on monitoring costs is dependent on the actual occurrence of prospective memory targets. In one condition, the participants were instructed for the prospective memory task, but no targets occurred, whereas in another condition the targets actually occurred. The prospective memory task was to press a key whenever one of eight specific target words occurred, embedded in a lexical decision task (i.e., a cognitive “loaded” prospective memory task). The results showed that importance improved prospective memory performance, but caused higher monitoring costs. Higher monitoring costs were also present in the condition in which the prospective memory task was instructed, but no prospective memory targets occurred, indicating that importance instructions enhanced strategic monitoring.

Smith and Hunt (2014) investigated age-effects of relative importance instructions. Prospective memory performance is normally impaired for older compared to young adults in laboratory research (cf. Zimmermann and Meier, 2006). This age-effect is assumed to be a result of the decreased cognitive resources which result in less strategic monitoring (see Kliegel et al., 2003; McDaniel et al., 2008a; Aberle et al., 2010; Altgassen et al., 2010). Smith and Hunt asked their participants to press a designated key whenever one of three possible prospective memory target words appeared. These words were embedded in an ongoing color-matching task. Additionally, half of the young adults group and half of the older adults group were instructed that the prospective memory task would be more important than the ongoing color-matching task whereas the other half were instructed that the color-matching task would be more important than the prospective memory task. Results showed that the younger adults outperformed the older adults in the prospective memory task. Moreover, the groups in the prospective memory task importance condition outperformed the groups in the ongoing color-matching task importance condition. There was no interaction between age and importance manipulation. This suggests an age-related decline of prospective memory performance, a comparable effect of importance across age groups and comparable monitoring costs for younger and older adults.

In contrast, Hering et al. (2013) found that younger adults were not influenced by a relative importance manipulation. They asked a group of young and a group of older participants to work with a two-back working memory task as an ongoing task and to press a designated key when one of six prospective targets occurred (i.e., a cognitive “loaded” prospective memory task). The older adults showed similar prospective memory performance as younger adults when the prospective memory task was emphasized, but the reduction of the age-effect came at an increased monitoring cost for older adults.

In summary, studies with relative importance manipulations typically showed enhanced prospective memory performance. Moreover, this increased performance came at costs in the ongoing tasks (but see Kliegel et al., 2001, 2004; Hering et al., 2013).

### Absolute importance manipulation

So far, only one study has investigated the impact of importance on prospective memory performance by simply emphasizing the importance of a prospective memory task vs. a control condition with standard instructions, that is, *absolute importance* (Einstein et al., 2005). In contrast to relative importance, this kind of importance manipulation does not explicitly stress dual-task prioritizing.

In this study, Einstein et al. (2005, Experiment 1) also compared a focal and a non-focal prospective memory cue condition (i.e., *concurrent overlap*, see Meier and Graf, 2000). The ongoing task was a word-categorization task and the prospective memory task was to press a particular key whenever a specific word (focal condition) or a word with the syllable “tor” appeared (non-focal condition). The results showed that stressing importance influenced prospective memory performance only in the non-focal condition. Consistently, monitoring costs were also increased in the latter condition. However, prospective memory performance in the focal condition was close to ceiling and thus the lack of an importance effect must be interpreted with caution.

### Providing social motives

Providing social motives to perform a prospective memory task (i.e., instructing participants an intention is important for somebody else) can also increase the importance of a task. This manipulation can be considered as a special case of an absolute importance manipulation. However, in addition, an explicit reason is given why the intention is important.

Using a naturalistic task in a laboratory context, Kvavilashvili (1987, Experiment 2) asked participants to perform a prospective memory task either after a filled or an unfilled retention interval (i.e., an *activity-based task*). Importance was induced by telling half of the participants that an important telephone call for the experimenter was waiting (i.e., social motive). The other half, the control group, was not given this additional information. The prospective memory task was to remember to hang up a telephone receiver that was suspended during the experiment. In addition, in the retention interval, participants were either engaged in an interesting activity, a monotonous activity, or were told to relax. The results showed that only very few participants forgot to perform the “important” prospective memory task, independent of the retention interval manipulation. In contrast, for participants in the control group forgetting was much more likely when an interesting activity was performed. In fact, Cicogna and Nigro (1998) replicated this finding with a very similar set-up. The only difference was that they asked participants to hang up the telephone after 5 min and then to return to the ongoing task which turned the prospective memory task into a time-based prospective memory task. Thus, it seems that the importance of an intention can shield prospective memory against distractions for both, activity-based and time-based prospective memory tasks.

Brandimonte et al. (2010) investigated the influence of providing a social motive and also its interaction with providing a reward. Participants were either told that their results would offer information that was particularly important to the researcher (i.e., social motive condition), that they would receive course credits if they remembered to carry out the prospective memory task appropriately (i.e., reward condition), both of these instructions (social motive and reward condition) or none of this information (i.e., standard prospective memory condition). The ongoing task was to decide whether a verb was regular or irregular. The prospective memory task was activity-based, namely, to sign a form at the end of each experimental block. The results showed that prospective memory task performance was better in the social motive condition compared to both the standard instruction condition and the reward condition. Astonishingly, prospective memory performance was lower when both a social motive and a reward were present. There were no monitoring costs in any of the conditions. Thus, the results of this study suggest that prospective memory performance can be improved by emphasizing the social importance of the task without increasing monitoring.

Altgassen et al. (2010) investigated age-effects of providing a social motive on prospective memory and ongoing task performance in a time-based prospective memory task. Participants were engaged in an ongoing visuo-spatial working-memory task and they had the additional instruction to press a designated key every 2 min as the prospective memory task. Moreover, half of the participants received the standard prospective memory task instruction (i.e., control group) whereas the other half of the participants received the social importance instruction. In the latter, they were told that they were doing the experimenter a favor when they would remember to press the key after the 2 min time-periods. Results showed that younger adults generally outperformed older adults in the prospective memory task. However, there was an interaction between age-group and importance manipulation, which showed that younger adults were not influenced by the importance manipulation. In contrast, older adults improved prospective memory performance in the social importance instruction compared to the control group. Critically, this improvement neither did come at costs in the ongoing task nor at an increased time-checking behavior.

In summary, the provision of a social motive to produce importance also enhanced prospective memory performance in all of the reviewed studies. Critically, this did not come at a cost in the ongoing task (cf. Altgassen et al., 2010; Brandimonte et al., 2010).

## Underlying mechanisms of task importance

**Figure 1:**
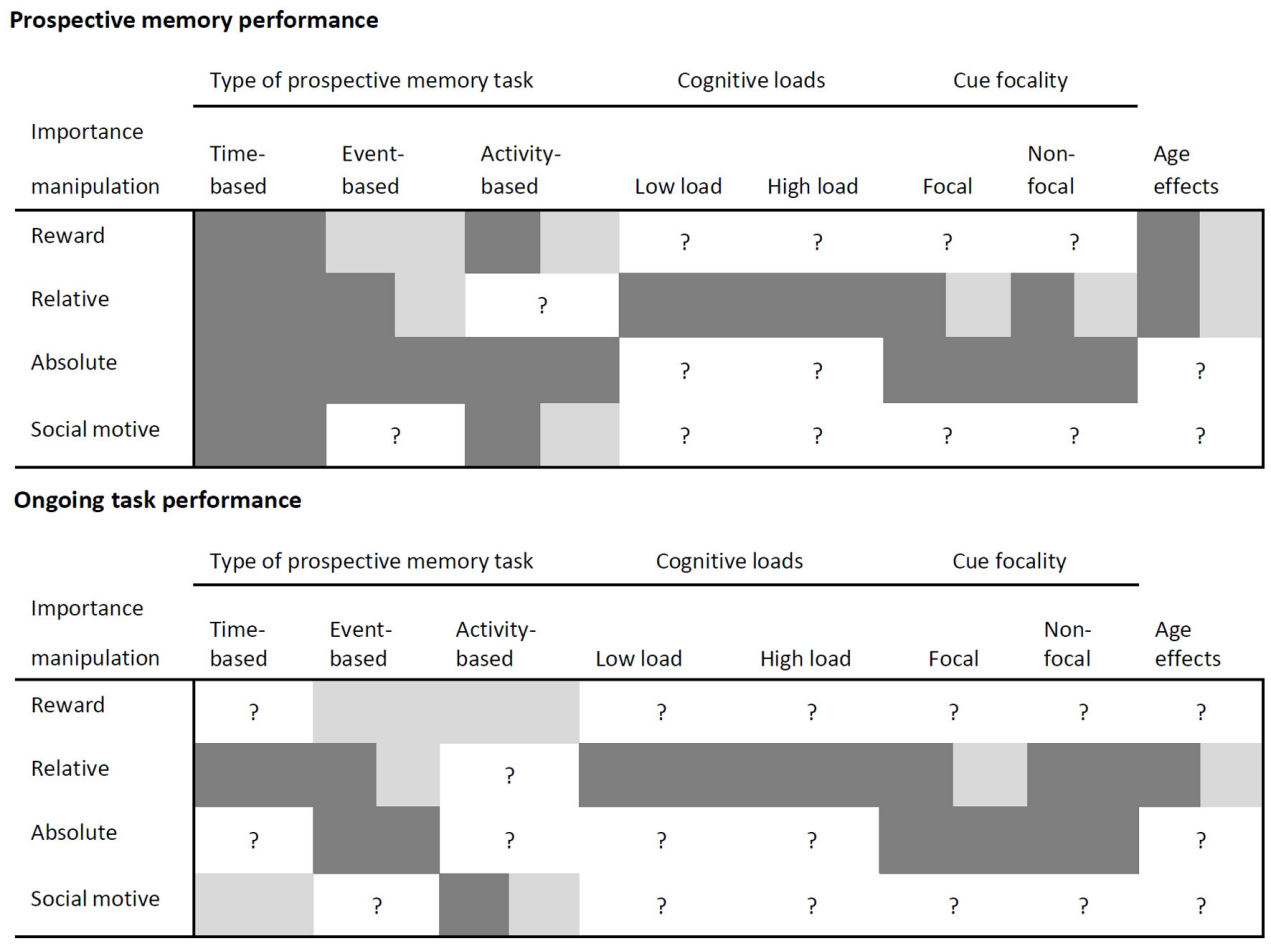
**Effects of importance on prospective memory performance and on ongoing task performance depending on the kind of importance manipulation, type of prospective memory task, cognitive loads, cue focality, and age**. Dark gray fields show enhanced prospective memory task performance or ongoing task costs, light gray fields show no changes in prospective memory task performance or ongoing task costs and white fields show knowledge gaps.

Most of the results of the reviewed studies showed that the importance of an intention increases prospective memory performance (cf. Meacham and Singer, 1977; Kvavilashvili, 1987; Einstein et al., 2005). However, some studies suggest that this is not necessarily the case and that other factors also influence prospective memory performance (Kliegel et al., 2001, 2004; Loft and Yeo, 2007; Brandimonte et al., 2010). Furthermore, enhancing prospective memory performance due to an importance manipulation sometimes resulted in costs for the ongoing task (e.g., Loft and Yeo, 2007; Smith and Hunt, 2014), but in a few studies, there was no indication for costs (e.g., Brandimonte et al., 2010). Consequently, we have to take into account differences regarding the type of prospective memory task, cognitive load, and potential processing overlaps. These factors might modulate the impact of task importance on prospective memory and ongoing task performance. The results of the reviewed studies including these factors are summarized in Figure 1.

Next, we will discuss the results of the reviewed studies from the perspective of the *PAM theory* and the *multiprocess model* in order to underpin this assumption. According to PAM, successfully remembering to perform a prospective memory task always comes at a cost in the ongoing task, because “preparatory attentional processes” are required to detect a prospective memory target. This induces a change in resource allocation policies to the prospective memory task through strategic monitoring (Smith and Bayen, 2004; Smith and Hunt, 2014; but see Meier and Rey-Mermet, 2012). This theory predicts that when an intention is important increased prospective memory performance is due to a change in resource allocation policies and thus, to increased strategic monitoring.

In contrast, according to the multiprocess model, there are two different routes toward remembering a prospective memory task (see McDaniel and Einstein, 2000; Einstein et al., 2005). Similar to PAM, prospective memory may come at a cost in the ongoing task due to strategic monitoring if, for example, a prospective memory target is not distinctive or if the association or the underlying processes between a target and the intended actions are weakly associated. In contrast, *automatic retrieval* of an intention without ongoing task costs is possible, if, for example, a prospective memory target is distinctive or with high processing overlaps (e.g., for focal cues).

Inducing importance may also lead to a *higher activation level* of the intention, to a *higher sensitivity* toward a prospective target event, and thus to *higher accessibility* of the prospective task. This could be similar to those metacognitive strategies which are also used to enhance prospective memory performance such as implementation intentions, imagery of a prospective memory task, or performance predictions (e.g., Gollwitzer, 1999; McDaniel et al., 2008b; Meeks and Marsh, 2009; Zimmermann and Meier, 2010; Brewer et al., 2011; Grilli and McFarland, 2011; McFarland and Glisky, 2011; Meier et al., 2011; Schult and Steffens, 2011; Rummel et al., 2012).

In light of these considerations, we suggest that inducing importance by providing a social motive or by an absolute importance instruction (i.e., importance instructions without an explicit request to prioritize the prospective memory task) may operate by similar mechanisms as metacognitive strategies, that is, they enhance the prospective memory *task-context associations* to perform a prospective memory task without increasing strategic monitoring. According to the multiprocess model, in some cases, prospective memory performance is enhanced due to a change in resource allocation policies (e.g., when producing importance by providing a reward depending on prospective memory performance or by a relative importance manipulation), but in other cases it is enhanced due to automatic retrieval (e.g., when producing importance by providing a social motive or by an absolute importance manipulation).

### Change in resource allocation policies vs. automatic retrieval

The results of the reviewed studies with relative importance manipulations showed that prioritizing the prospective memory task enhanced prospective memory performance, but this came at a cost in the ongoing task. Prioritizing the ongoing task reduced the cost compared to prioritizing the prospective memory task, but it also resulted in lower prospective memory task performance (see Marsh et al., 1998, for a detailed discussion of this issue).

**Figure 2:**
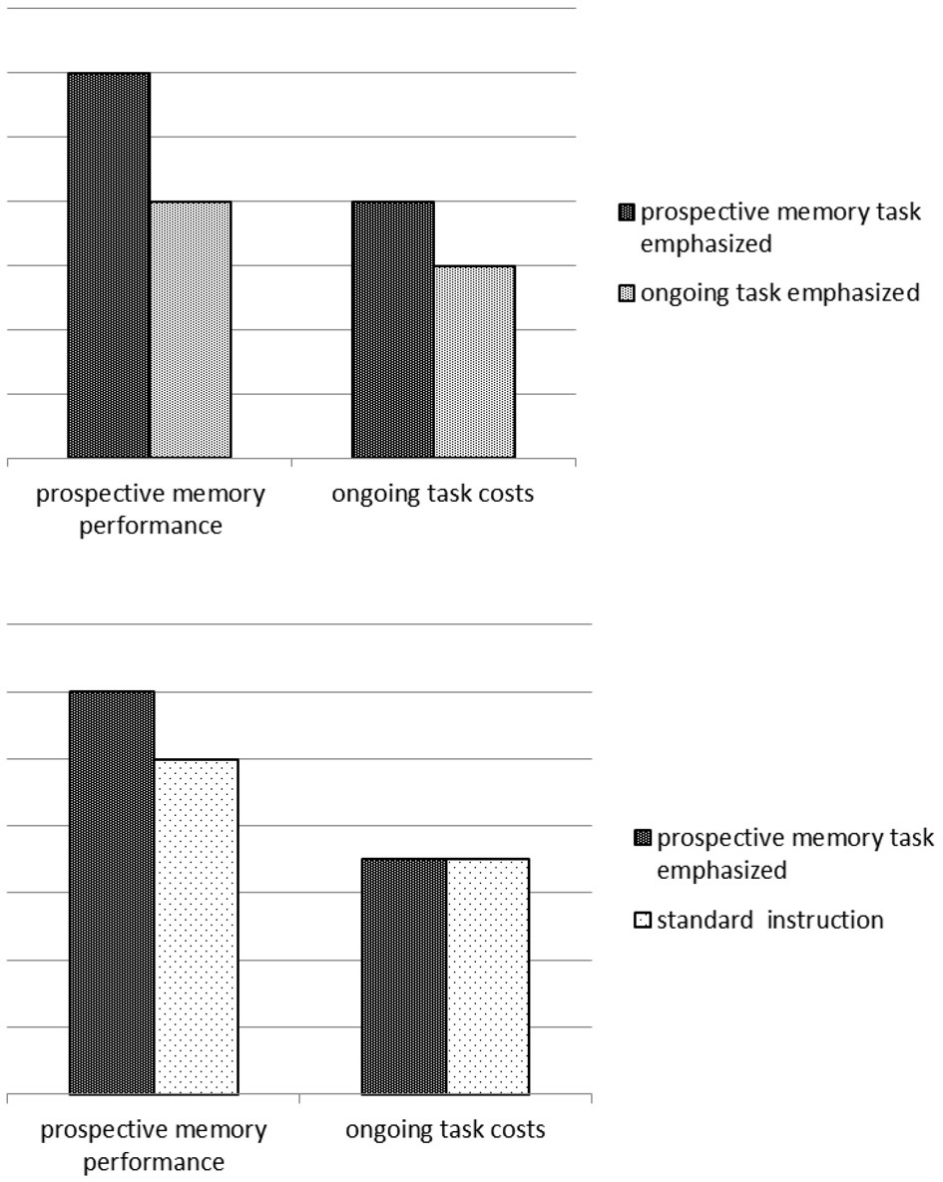
**Prospective memory performance and ongoing task costs for studies on relative performance (top) and possible outcome for absolute importance manipulations (bottom)**.

However, importantly, the studies that have investigated the effect of relative importance have not included a control condition, in which neither the importance of the prospective memory task nor the ongoing task is emphasized. Such a control condition would be necessary to determine whether an ongoing task cost exists at all, that is, over and above the cost that is typically associated with performing a laboratory prospective memory task (see Figure 2 for an illustration).

The results of the laboratory studies using a reward to produce importance suggested a change in resource allocation policies. However, in most of these studies monitoring costs were not measured, or in one study only a subjective rating was used (Krishnan and Shapiro, 1999). Moreover, there are two studies in which monitoring costs were measured (Brandimonte et al., 2010; Kliegel et al., 2010), but no monitoring costs occurred. Critically, both of these studies involved activity-based prospective memory tasks. Thus, the type of prospective memory task may modulate the effect of an importance manipulation.

The results of studies with absolute importance instructions showed that prospective memory task performance was increased compared to standard prospective memory task instruction and this increase came at a cost in the ongoing task. This suggests that the retrieval of an important intention may be based on a change of resource allocation policies, also for absolute importance. Nevertheless, so far, solid evidence for the question whether absolute importance also affects ongoing task performance is lacking, because the instruction of the only study that used a proper absolute importance instruction explicitly pushed participants to find every cue and may thus have induced strategic monitoring (cf. Einstein et al., 2005). Further research is necessary to follow up on this result with less suggestive instructions.

Importantly, with absolute importance instructions, participants are not directly prompted to prioritize one task over the other. There is no explicit reason to change resource allocation policies to the prospective memory task. Therefore, absolute importance instructions could also enhance prospective memory performance due to automatic retrieval and critically would not necessarily come at costs in the ongoing task (see Figure 2 for an illustration). Future studies are necessary to test this hypothesis.

The results of studies using social motives as importance manipulation showed increased prospective memory performance without costs in the ongoing task. This supports the assumption that automatic retrieval can occur due to an enhanced association between a prospective memory target and the prospective memory task (Altgassen et al., 2010; Brandimonte et al., 2010). The results of the seminal study by Kvavilashvili (1987) are in line with this interpretation, because communicating to participants that the experimenter would be waiting for an important call may have also enhanced the social motive. Moreover, the results of the study by Brandimonte et al. (2010) that prospective memory performance was enhanced when people had a social motive to remember the intention, but not when additionally a reward was provided is thought-provoking. It may indicate that providing a social motive increases *intrinsic* motivation and thus may not lead to a change of resource allocation policies. However, providing a reward over and above a social motive could affect *extrinsic* motivation (e.g., Brandimonte and Ferrante, 2008) and change the attention allocation policy. We will elaborate on this motivational account of prospective memory performance in the next section.

## A motivational account of importance manipulations

The impact of emphasizing importance, providing a reward and activating a social motive may have unique motivational consequences because importance is based on a subjective evaluation of personal goals and predicted consequences (e.g., Goschke and Kuhl, 1996; Kvavilashvili and Ellis, 1996; Penningroth and Scott, 2007; Altgassen et al., 2010; Brandimonte et al., 2010; Meier et al., 2011; D'Angelo et al., 2012; Niedźwieńska et al., 2013). Two aspects of motivation can be distinguished, intrinsic and extrinsic (Deci and Ryan, 1985). Intrinsic motivation refers to an inherent interest in performing a particular task. In contrast, extrinsic motivation refers to a means-end interest in performing a task. We propose that activating a social motive is more likely to enhance intrinsic motivation, whereas providing a (monetary) reward is more likely to enhance extrinsic motivation (cf. Bear et al., 2003; Savine et al., 2010). Moreover, we suggest that the type of motivation is directly related to potential changes in attention allocation policies. In studies using a reward or a relative importance instruction, importance effects are accompanied by costs in the ongoing task (e.g., Kliegel et al., 2004; Smith and Bayen, 2004). Thus, these importance manipulations increase extrinsic motivation and result in a change of attention allocation (cf. Delgado et al., 2003; Savine et al., 2010).

In contrast, importance manipulations such as providing a social motive and, potentially, absolute importance manipulations enhance intrinsic motivation and this may strengthen the representation of the prospective memory task rather than change the attention allocation policy (cf. Ślusarczyk and Niedźwieńska, 2013). Thus, intrinsic motivation may enable the automatic retrieval of a prospective memory task and therefore importance manipulations must not necessarily be accompanied by a cost in the ongoing task performance (e.g., Penningroth and Scott, 2007). It is possible that for these kinds of importance manipulations similar mechanisms may be at work as for other manipulations used to enhance prospective memory performance such as implementation intentions, imagery of a prospective memory task, or performance predictions (e.g., Gollwitzer, 1999; McDaniel et al., 2008b; Meeks and Marsh, 2009; Zimmermann and Meier, 2010; Brewer et al., 2011; Grilli and McFarland, 2011; McFarland and Glisky, 2011; Meier et al., 2011; Schult and Steffens, 2011; Rummel et al., 2012).

This hypothesis is also supported by neuropsychological findings. Providing a reward increased activation in prefrontal regions which support the control and active maintenance of task-goals and may therefore increase strategic monitoring (e.g., Braver, 2012). In contrast, metacognitive strategies are based on temporal networks known to support memory encoding and retrieval (e.g., Gordon et al., 2011). Similarly, monitoring in a prospective memory task is associated with increased prefrontal cortex activation whereas automatic retrieval is associated with increased temporal cortex activation (cf. McCauley et al., 2009; McDaniel et al., 2013). This can be taken as further evidence that importance effects are based on different routes toward remembering which may be driven by extrinsic or intrinsic motivation.

These considerations about motivation also have important consequences for interventions to reduce age-related differences. Specifically, it has been suggested that older adults are more prone to intrinsic motivation (Deci and Ryan, 1985). Thus, manipulations that aim at increasing intrinsic motivation may enhance prospective memory performance without interfering with the ongoing task. However, this needs also further investigations.

To summarize, whether a particular importance manipulation affects resource allocation policies may depend on whether motivation is extrinsic. Respectively, whether automatic retrieval is the underlying mechanism may depend on whether motivation is intrinsic. However, the relationship between different importance manipulations and motivation needs further investigation to disentangle the underlying mechanisms of task importance (see also Penningroth and Scott, 2007, for a similar assumption).

## Conclusion

The purpose of this article was to review the literature on the impact of importance on prospective memory performance. We have identified several different importance manipulations such as providing rewards, relative importance instructions, absolute importance instructions, and providing a social motive. We have also focused on whether importance manipulations affect resource allocation polices or not—that is whether strategic monitoring is necessary or whether automatic retrieval is sufficient. Our review shows that, in general, prospective memory performance is enhanced by importance and that ongoing task costs show a differential pattern for the different importance manipulations.

Most importantly, our review suggests that for providing rewards and relative importance instructions a change in resource allocation policies occurs and thus, strategic monitoring is used to perform the prospective memory task. In contrast, for providing social motives and absolute importance instructions, rather automatic retrieval is the basis for successful performance. Our motivational account supports this assumption. According to this account, intrinsic and extrinsic motivation are directly related to importance manipulations and while extrinsic motivation induces strategic monitoring, intrinsic motivation enhances the activation of intention representation and leads to a performance advantage due to automatic retrieval.

We have identified many open questions, which call for a more systematic investigation of importance effects in prospective memory. First, more work is needed to clarify the relation between motivation, importance and their underlying mechanisms. Second, as can be seen in Figure 1, there are many combinations of factors that have been addressed in this review, but have not yet been studied empirically. Future studies should systematically investigate the influence of the impact and the interplay of type of importance manipulation, type of prospective memory task, cognitive loads, and processing overlaps on prospective memory as well as on ongoing task performance (see also Supplementary Table 1). Finally, studies investigating the influence of relative importance on prospective memory should also consider a control condition without an importance instruction to clarify the contribution of task importance to changes in resource allocation policies. Altogether, we have identified important information lacunas which can guide future investigations on the effects of importance on prospective memory and on ongoing task performance in order to supply importance effects with a higher economic validity.

Last but not least, we would like to get back to the question raised in the title of this article. Our review shows that importance *is* important as it typically increases prospective memory performance. Moreover, it can affect ongoing task costs dependent on the type of motivation triggered (i.e., intrinsic or extrinsic). In laboratory research, the methods to produce importance can be considered as “planning guidelines.” In daily life, the different importance of our multiple intentions allow us to prioritize and as a consequence to flexibly select our future goals.

### Conflict of interest statement

The authors declare that the research was conducted in the absence of any commercial or financial relationships that could be construed as a potential conflict of interest.
