# Supplementary material for: How important is importance for prospective memory? A review
Source: Front Psychol. 2014 Jun 26;5:657. doi: 10.3389/fpsyg.2014.00657 (PMC4071817; doi:10.3389/fpsyg.2014.00657)
Supplement: Supplementary file 1 [file DataSheet1.DOCX]

Supplementary Table 1

|  |  | Importance manipulation | | | |  | | Type of task | | | | | | |  | | Cognitive  loads | | |  | | Cue focality  Processing overlaps | | | | | | |  | Importance effect | | | | |
| --- | --- | --- | --- | --- | --- | --- | --- | --- | --- | --- | --- | --- | --- | --- | --- | --- | --- | --- | --- | --- | --- | --- | --- | --- | --- | --- | --- | --- | --- | --- | --- | --- | --- | --- |
| Studies |  | Reward | Relative | Absolute | Social motive |  | Time-based | | | | Event-based | | Activity-based | |  | Type of load | | | | |  | Sequential overlap | | | Concurrent overlap | | Cue-content overlap | |  | Monitoring  costs | | | ProM performance | |
| Kliegel et al. (2001) Experiment 1 |  |  | X |  |  |  | X | | | |  | |  | |  |  | | | | |  |  | | |  | |  | |  | 🡩 | | | 🡩 | |
| Kliegel et al. (2001) Experiment 2 |  |  | X |  |  |  |  | | | | X | |  | |  | DB | | | | |  |  | | | F | |  | |  | 🡩 | | | -- | |
| Kliegel et al. (2004) Experiment 1 |  |  | X |  |  |  |  | | | | X | |  | |  | DB | | | | |  |  | | | F | |  | |  | 🡩 | | | -- | |
| Kliegel et al. (2004) Experiment 2 |  |  | X |  |  |  |  | | | | X | |  | |  | DB | | | | |  |  | | | NF | |  | |  | 🡩 | | | 🡩 | |
| Smith & Bayen (2004) |  |  | X |  |  |  |  | | | | X | |  | |  | NT | | | | |  |  | | | NF | |  | |  | 🡩 | | | 🡩 | |
| Loft & Yeo (2007) Experiment 2 |  |  | X |  |  |  |  | | | | X | |  | |  | NT | | | | |  | (F) | | |  | | F; NF | |  | 🡩 | | | 🡩 | |
| Loft et al. (2008)  Experiment 2 |  |  | X |  |  |  |  | | | | X | |  | |  | NT | | | | |  | (F) | | |  | |  | |  | 🡩 | | | 🡩 | |
| Harrison & Einstein (2010) |  |  | X |  |  |  |  | | | | X | |  | |  |  | | | | |  |  | | |  | |  | |  | -- | | | -- | |
| Smith (2013)  Experiment 1 |  |  | X |  |  |  |  | | | |  | |  | |  |  | | | | |  |  | | |  | |  | |  | 🡩 | | | 🡩 | |
| Hering et al.(2013) |  |  | X |  |  |  |  | | | | X | |  | |  | NT | | | | |  |  | | |  | |  | |  | 🡩 only for older adults | | | 🡩 only for older adults | |
|  |  |  |  |  |  | | | |  |  | |  | |  | | | |  |  | | | |  |  | |  | |  | | |  |  | | (continued) |

Overview of prospective memory performance and ongoing task costs of studies investigating the importance of intentions varying importance manipulations, types of tasks, cognitive loads and cue focality.

|  | Importance manipulation | | | |  | Type of task | | |  | | Cognitive  loads | |  | | Cue focality  (processing overlaps) | | |  | Importance effect | |
| --- | --- | --- | --- | --- | --- | --- | --- | --- | --- | --- | --- | --- | --- | --- | --- | --- | --- | --- | --- | --- |
| Studies | Reward | Relative | Absolute | Social motive |  | Time-based | Event-based | Activity-based | |  | | Type of load | |  | Sequential overlap | Concurrent overlap | Cue-content overlap |  | Monitoring costs | ProM Performance |
| Einstein et al. (2005) |  |  | X |  |  |  | X |  | |  | | NT | |  | (F) | F; NF |  |  | 🡩 | 🡩 |
| Kvavilashvili (1987) Experiment 2 |  |  | (X) | X |  | X |  | (X) | |  | | (I) | |  |  |  |  |  | ? | 🡩 |
| Cicogna & Nigro (1998) |  |  | (X) | X |  | X | X |  | |  | |  | |  |  |  |  |  | ? | 🡩 |
| Krishnan & Shapiro (1999) | X |  |  |  |  |  | X |  | |  | |  | |  |  |  |  |  | 🡩 | 🡩 |
| Gujardo & Best (2000) | X |  |  |  |  |  | X |  | |  | |  | |  |  |  |  |  | -- | -- |
| Kliegel et al. (2003) | X |  |  |  |  |  | X |  | |  | |  | |  |  |  |  |  | ? | -- |
| McCauley et al. (2011) | X |  |  |  |  |  | X |  | |  | |  | |  |  |  |  |  | ? | 🡩 |
| Meacham & Singer (1977) | X  (+A) |  |  |  |  | X |  |  | |  | |  | |  |  |  |  |  | ? | 🡩 |
| Jeong & Cranney (2009) | X |  |  |  |  | X |  |  | |  | |  | |  |  |  |  |  | ? | 🡩 |
| Aberle et al. (2010)  Experiment 2 | X |  |  |  |  | X |  |  | |  | |  | |  |  |  |  |  | ? | 🡩 only for young adults |
| Brandimonte et al. (2010) | X |  |  | X |  |  |  | X | |  | |  | |  |  |  |  |  | -- | 🡩 with social benefit  no effect of rewards |
| D’Angelo et al. (2012)  Experiment 2 | X |  |  | X |  |  |  |  | |  | |  | |  |  |  |  |  | 🡩 with social benefit | 🡩 with social benefit  no effect of rewards |
| Altgassen et al. (2010) |  |  |  | X | X |  |  |  | |  | |  | |  |  |  |  |  | -- | 🡩 only for older adults |
| Sommerville et al. (1983) | A |  |  |  |  | X | X | X | |  | |  | |  |  |  |  |  | ? | 🡩 |
| Kliegel et al. (2010) | A |  |  |  |  |  |  | X | |  | |  | |  |  |  |  |  | -- | 🡩 |
| Ślusarczyk & Niedźwieńska (2013) | A |  |  |  |  |  |  |  | |  | |  | |  |  |  |  |  | -- | 🡩 |

*Note.* ProM = prospective memory; A = attractiveness of rewards or tasks; OTL = ongoing task load; EB = event-based; DB = demanding background; NT = high number of targets; I = Involvement; F = focal (i.e. high processing overlap); NF = non focal (i.e. low processing overlap); 🡩 = effect of importance; ? = no analysis
